# Morvan syndrome associated with LGI1 antibody: a case report

**DOI:** 10.1186/s12883-021-02205-9

**Published:** 2021-05-03

**Authors:** Shui-Jing Zhang, Yan-Yan Xue, Hao Yu, Qing-Qing Tao

**Affiliations:** 1 Department of Neurology,The Third Affiliated Hospital of Zhejiang Chinese Medical University and Zhejiang Rehabilitation Medical Center, Hangzhou, China; 2 Department of Neurology and Research Center of Neurology in Second Affiliated Hospital, Key Laboratory of Medical Neurobiology of Zhejiang Province, Zhejiang University School of Medicine, Hangzhou, China

**Keywords:** Morvan syndrome, LGI1, CASPR2, Neuromyotonia

## Abstract

**Background:**

Morvan syndrome (MoS) is a rare autoimmune syndrome associated with antibodies against two kinds of potassium channel proteins, contactin associated protein-like 2 (CASPR2) and leucine-rich glioma inactivated protein 1 (LGI1). MoS patients with only LGI1-antibody seropositivity have rarely been reported. Here, we describe a 64-year-old male MoS patient with only LGI1-antibody seropositivity.

**Case presentation:**

A 64-year-old male patient was referred to our hospital due to limb pain, widespread myokymia, insomnia, constipation, and hyperhidrosis for 1 month. The patient was diagnosed with MoS based on the clinical symptoms and positive LGI1-antibody in serum. He was treated with intravenous immunoglobulin (IVIG), intravenous methylprednisolone followed by oral prednisone, and other drugs for symptomatic relief. Several days later, myokymia and insomnia symptoms improved. After 60 days of follow-up, all the drugs had been stopped for 2 weeks, and the patient achieved complete remission without any medical side effects.

**Conclusion:**

We report the clinical characteristics of a Chinese MoS patient with only LGI1-antibody seropositivity, and further support the view that non-neoplasm MoS patients respond well to immunotherapy.

**Supplementary Information:**

The online version contains supplementary material available at 10.1186/s12883-021-02205-9.

## Background

Morvan syndrome (MoS) is a rare autoimmune syndrome associated with antibodies against two kinds of potassium channel proteins, contactin associated protein-like 2 (CASPR2) and leucine-rich glioma inactivated protein 1 (LGI1) [1]. In the last decades, a number of MoS patients with only CASPR2 antibody (CASPR2-Ab) seropositivity or both CASPR2-Ab and LGI1 antibody (LGI1-Ab) seropositivity have been successively reported worldwide [2, 3]. However, MoS patients with only LGI1-Ab seropositivity have rarely been reported. Here, we first describe the clinical features of a MoS patient with only LGI1-Ab seropositivity in the Chinese population, who had a good response to immunotherapy.

## Case presentation

A 64-year-old male patient presented with limb pain, widespread myokymia, insomnia, constipation, and hyperhidrosis for 1 month. Two days before the disease onset, he had a fever with a maximum temperature of over 39 °C and was treated with antipyretic and antibacterial drugs at a local hospital. Though recovering from the fever, he began to feel limbs pain, accompanied by myokymia, insomnia, constipation, and hyperhidrosis. Constipation and hyperhidrosis were relieved 1 week later. However, limb pain, widespread myokymia, and insomnia still afflicted him. The neurological examinations showed a normal mental status. Examinations of the cranial nerves were unremarkable. Myokymia could be seen in the trunk and bilateral limbs (Supplemental materials). Muscle strength, tendon reflexes, and sensory examinations were normal. He had no remarkable past medical history, personal history, or family history.

**Fig. 1:**
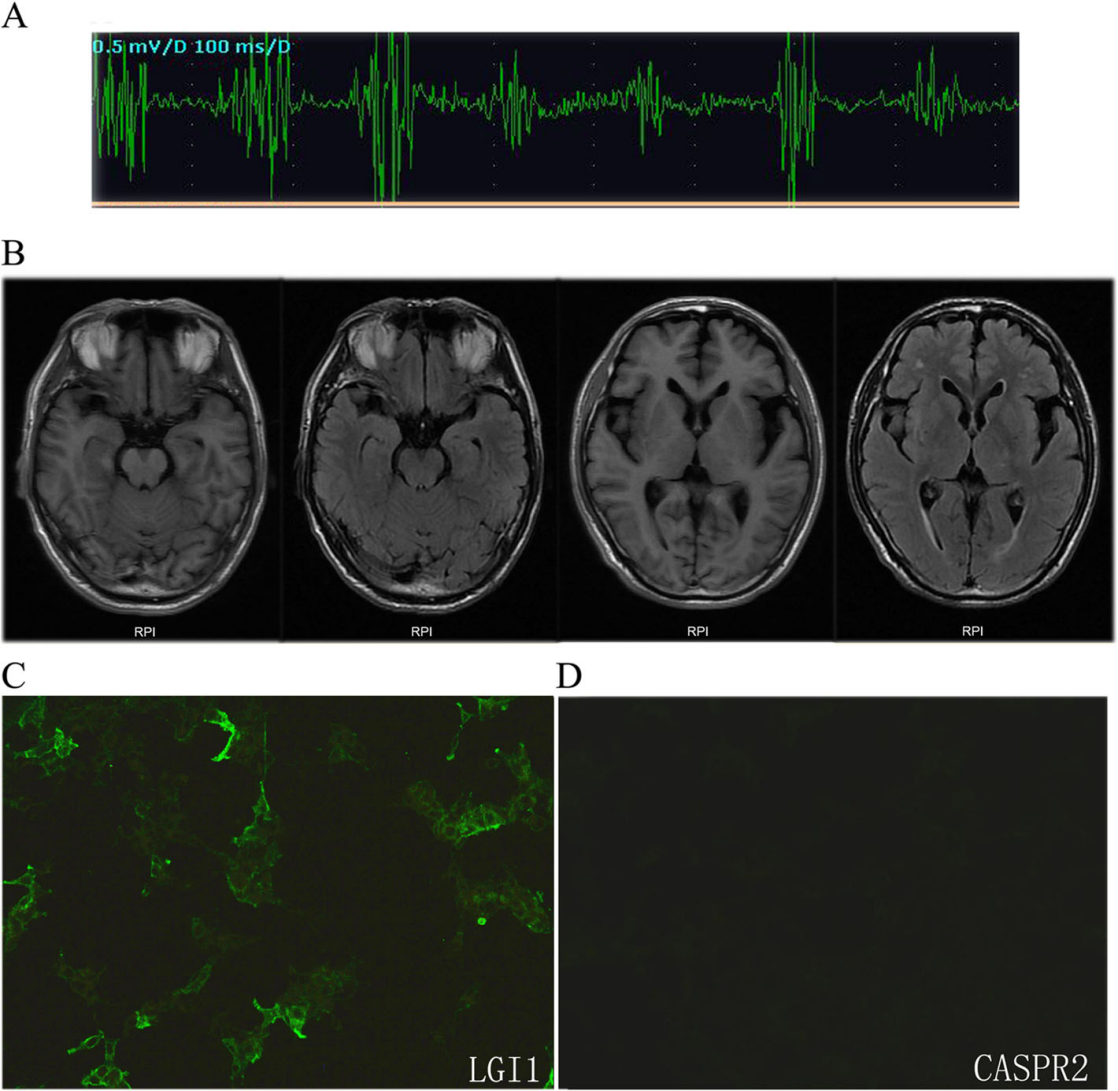
Clinical findings in the MoS patient with LGI1 antibody**. a** Needle EMG showed grouped discharges on the right gastrocnemius muscles. **b** The brain MRI examination showed no obvious abnormalities. **c**, **d** The immunoreactivity of patient’s serum to LGI1 and CASPR2 proteins was examined by the indirect immunofluorescence test (IIFT)

The routine blood tests, including full blood count, hematocrit, liver, kidney and thyroid functions, disclosed unremarkable findings. The patient’s muscle enzymes were normal. Tumor markers (CEA, AFP, CA125, CA153, CA199, CA724, NSE, and PSA) were all negative. Electromyography (EMG) showed muscle fiber twitch potential in bilateral limbs (Fig. 1a), with no abnormalities in sensory and motor nerve conduction. The brain MRI examination showed no abnormalities (Fig. 1b). The serum test using cell-based assay (EUROIMMUN, Germany) performed in a local testing agency showed LGI1-Ab was positive (+), while CASPR2-Ab, AMPA1 and AMPA2 antibodies were all negative (−) (Fig. 1c, d). Twenty-four-hour video-polysomnography revealed rapid eye movement (REM) sleep latency was 221 min and REM accounted for 8.6% of total sleep time. Twenty seven awakenings happened during the whole sleep process, and the awakening time was 115.8 min in total. Chest and abdomen CT scanning showed no malignancy, but the patient refused positron emission tomography (PET) examination. Before we got the serum antibody results, to rule out certain types of myopathy, the gastrocnemius muscle biopsy was conducted and showed no abnormality.

The patient was diagnosed with MoS based on the combination of myokymia, insomnia, and dysautonomia associated with positive LGI1-Ab and typical EMG performance. He was treated with intravenous immunoglobulin (IVIG) at a dose of 0.4 g/kg/day for 5 days and intravenous methylprednisolone 120 mg/day for 3 days, followed by oral prednisone 60 mg/day with gradual tapering over a period of a month. Insomnia was treated with clonazepam 2 mg 30 min prior to bedtime, and limb pain with gabapentin 0.3 g three times a day combined with duloxetine 60 mg/day. Ten days later, symptoms of myokymia and insomnia improved (Supplemental materials), but limb pain was not relieved. Gabapentin was then replaced by pregabalin 150 mg twice a day. During 30 days’ follow-up, limb pain got significantly relieved. At 60 days’ follow-up, when all the drugs had been stopped for 2 weeks, the patient achieved complete remission. According to 6-month follow-up results, the patient showed no obvious symptoms of discomfort.

## Discussion and conclusions

In 1890, the French physician Augustine Marie Morvan first described a disease characterized by autonomic dysfunction and severe insomnia, which known as Morvan’s syndrome now. So far, less than 100 MoS cases have been reported [4]. A wide variety of clinical symptoms, including myokymia, autonomic nerve symptoms, insomnia accompanied by encephalopathy have been reported [5, 6]. MoS is now considered as one of the peripheral nerve hyperexcitability (PNH) syndromes, which causes widespread symptoms and signs without the association of evident peripheral nerve disease, and mainly manifests muscle twitching and stiffness [7].

MoS belongs to the broad category of autoimmune neurological disorders, primarily associated with antibodies against two kinds of potassium channel proteins, CASPR2 and LGI1 [1]. Various combinations of LGI1-Ab and CASPR2-Ab are associated with different phenotypes of MoS, which could be explained by the different distribution of targeted antigens [2]. LGI1 is expressed in both the peripheral and central nervous systems [8], while CASPR2 is expressed mainly in the peripheral nervous system [5]. LGI1-Ab is usually linked with limbic encephalitis (LE), faciobrachial dystonic seizures, hyponatremia, and REM sleep behavior disorders [9]. CASPR2-Ab tends to cause nerve rigidity and MoS [10]. LGI1-Ab may affect synaptic signal transduction, resulting in increased neuronal excitability [11, 12]. Antibodies in MoS patients’ serum are usually directed against LGI1, CASPR2, or both, but CASPR2-Ab dominates [2, 13].

Brain MRI and PET scan of MoS cases are usually normal, while electroencephalogram (EEG) sometimes shows diffuse slow waves, occasional 4–6 Hz theta wave activity, and a typical rapid eye movement (REM) with absence of non-rapid eye movement sleep (NREM) stage. Electromyography is useful in diagnosis of MoS, which is characterized by spontaneous myofiber activity with various denervation potentials [2, 14]. MoS with only LGI1-Ab seropositivity is classified as MoS1 and has the similar symptoms as MoS with both CASPR2-Ab and LGI1-Ab seropositivity, including myokymia, sleep disturbance, dysautonomia; however, referring to the previous literature and our findings, MoS with both CASPR2-Ab and LGI1-Ab seropositivity showed a correlation with thymomas, while MoS with only LGI1-Ab seropositivity did not [2]. The main diagnosis basis of MoS is clinical manifestations [2, 15], and our patient was diagnosed with MoS based on the combination of peripheral nerve hyperexcitability (referring to myokymia), insomnia, dysautonomia associated with LGI1-Ab seropositivity and typical EMG performance. Irani et al. reported 29 MoS patients, of which three patients were only positive for LGI1-Ab [2]. All three patients manifested hallucinations, amnesia, peripheral neuropathy, confusion/disorientation, myokymia, and pain [2]. Previous studies reported that the majority of MoS patients without neoplasm responded well to immunotherapy [11]. It is not surprising that our patient experienced a complete remission at 60 days’ follow-up after immunotherapy, including IVIG and corticosteroids.

Besides, many MoS patients had a history of infection before the disease onset as happened in the present case. Some studies claimed that infection was a trigger for MoS, and relevant symptoms might be a delayed immune response [4]. It has been reported that 0–18% of patients with LGI1-Ab seropositivity will have a relapse within years after the onset of the initial disease [16–18].

MoS patients with only LGI1-Ab seropositivity were rarely reported. To the best of our knowledge, this is the first MoS case with only LGI1-Ab seropositivity in the Chinese population. Our results further support the view that non-neoplasm MoS patients respond well to immunotherapy. Considering some of the patients with LGI1-Ab will relapse, a longer follow-up may be more helpful in understanding MoS.

## Supplementary Information

**Additional file 1: Video 1**. Widespread myokymia was observed in all limbs and trunk before IVIG treatment.

**Additional file 2: Video 2**. Symptoms of myokymia were significantly improved after treatment with IVIG for 5 days.

## Data Availability

Not applicable.

## Abbreviations

MoS: Morvan syndrome
PNH: Peripheral nerve hyperexcitability
LGI1- Ab: Leucine rich glioma inactivated protein 1 antibody
CASPR2-Ab: Contactin associated protein-like 2 antibody
EMG: Electromyography
REM: Rapid eye movement
IVIG: Intravenous immunoglobulin
NMT: Neuromyotonia
LE: Limbic encephalitis
EEG: Electroencepalogram
NREM: Non-rapid eye movement sleep
